# Saffron (*Crocus sativus*) and its constituents in ovalbumin-induced asthma model: a preclinical systematic review and meta-analysis

**DOI:** 10.3389/fphar.2024.1436295

**Published:** 2024-09-04

**Authors:** Hassan Ghobadi, Farzin Aslani, Mohammad Hossein Boskabady, Saeideh Saadat, Mohammad Reza Aslani

**Affiliations:** ^1^ Lung Diseases Research Center, Ardabil University of Medical Sciences, Ardabil, Iran; ^2^ Department of Orthopedics, Imam Khomeini Hospital, Tehran University of Medical Sciences, Tehran, Iran; ^3^ Applied Biomedical Research Center, Mashhad University of Medical Sciences, Mashhad, Iran; ^4^ Department of Physiology, School of Medicine, Zahedan University of Medical Sciences, Zahedan, Iran

**Keywords:** asthma, saffron, crocin, safranal, Crocus sativus, ovalbumin, meta-analysis

## Abstract

**Background:**

Animal and human studies have demonstrated that the saffron and the active components of saffron, including crocin, crocetin, and safranal, possess anti-inflammatory, antioxidant, and immunomodulatory properties. In this meta-analysis, the preclinical evidence and potential mechanism of saffron were explored in an animal model of ovalbumin-induced asthma.

**Methods:**

Studies related to saffron and its constituents in an animal model of ovalbumin-induced asthma from the beginning to March 2024 were searched from Scopus, PubMed, and Web of Science databases. The methodological quality of the studies was evaluated using the 15-item CAMARADES checklist. Data analysis was performed using STATA software version 17.

**Results:**

Thirteen studies with 536 animals (268 animals in the intervention group and 268 animals in the ovalbumin-induced group) were analyzed. The meta-analysis findings demonstrated that saffron and its constituents played a significant role in reducing total WBC, eosinophil, lymphocyte, and monocyte counts. Moreover, saffron showed a significant decrease in the levels of IL-4, IL-5, IL-13, IgE, histamine, endothelin, nitric oxide, and nitrite. Moreover, saffron was found to elevate EC50 thresholds and lower maximum response rates in experimental animals. The analysis revealed a significant identification of modulation in endoplasmic reticulum (ER) stress markers and miRNAs pathways.

**Conclusion:**

Saffron and its components may impact ovalbumin-induced asthma model in animals through anti-inflammatory, antioxidant, and immunomodulatory pathways, as well as improving pulmonary function and modulating ER stress markers and miRNAs pathways. As a result, saffron should be considered for further clinical trials in individuals suffering from asthma.

## Graphical Abstract

**Figure:**
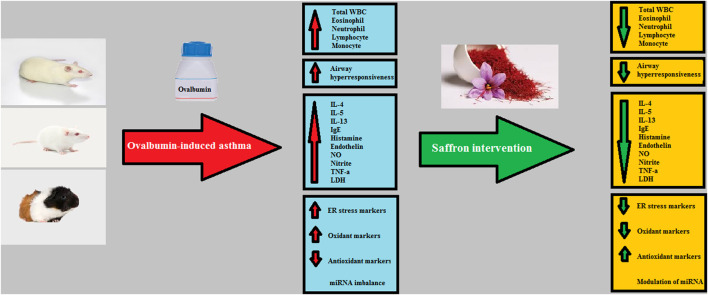

## 1 Introduction

Asthma is marked by airway hyperresponsiveness (AHR), increased infiltration of eosinophil and other inflammatory cells into the lungs, activation of T-helper two cells, higher levels of inflammatory cytokines (such as interleukin (IL)-4, IL-5, IL-13, IL-17, IL-1β, and IL-33), and noticeable histopathological changes (such as mucus hypersecretion, airway smooth muscle hypertrophy, and airway narrowing) (Aslani et al., 2016a; Aslani et al., 2016b; Athari, 2019). Animal models are increasingly utilized in basic research to enhance understanding of disease pathophysiology and develop novel treatment strategies. Sensitizing animals with ovalbumin (OVA) is a method frequently used to induce asthma in experiments (Keyhanmanesh et al., 2018; Ghobadi et al., 2019). The OVA-induced asthma model in animal studies has provided evidence of elevated inflammatory cells present in bronchoalveolar lavage fluid (BALF), modifications to lung histopathology, raised levels of inflammatory cytokines and mediators, enhanced AHR, and impairments to the oxidant-antioxidant balance (Akhavanakbari et al., 2019; Ghobadi et al., 2019; Warren et al., 2019).

Despite the use of multiple medications for asthma treatment, doubts have arisen regarding their efficacy, particularly in cases of severe and treatment-resistant asthma. Recently, attention to herbal medicines has been of interest in studies (Boskabady et al., 2007; Ghasemi et al., 2021; Khazdair et al., 2021; Saadat et al., 2021). Saffron is a plant that has been of interest for food and therapeutic uses in traditional medicine. Research has indicated that saffron has beneficial effects on various health conditions such as asthma (Abedi et al., 2023), chronic obstructive pulmonary disease (COPD) (Ghobadi et al., 2022a; Aslani et al., 2023a), polycystic ovary syndrome (PCOS) (Rahimi et al., 2022), diabetes (Sani et al., 2022), obesity (Mashmoul et al., 2013), metabolic syndrome (Shafiee et al., 2017), and cardiovascular disorders (Saadat et al., 2024) due to its active constituents such as crocin, crocetin, and safranal in both human trials and animal studies. The main function of saffron and its key ingredients is to offer defense against various conditions using anti-inflammatory, antioxidant, anticancer, and antiapoptotic pathways (Maqbool et al., 2022). Despite the positive impact of animal studies on scientific progress, the challenge of dealing with conflicting results remains. Saffron was one of the plant compounds included in the ovalbumin-induced asthma model, along with its active constituents. Therefore, the purpose of this study was to perform a comprehensive review and meta-analysis in the ovalbumin-induced asthma model to explore the protective properties of saffron, crocin, crocetin, and safranal.

## 2 Methods

The Preferred Reporting Strategies for Systematic Reviews and Meta-Analyses (PRISMA) guidelines served as the foundation for the conducted systematic review and meta-analysis.

### 2.1 Database and literature search strategies

Experimental studies evaluating the effects of saffron and its constituents (crocin, crocetin and safranal) in animal models of asthma (ovalbumin sensitization) were identified from PubMed, Web of Science, Science Direct, and Scopus by searching all published articles from the beginning to March 2024. Supplementary Table S1 contains the keywords that were utilized. The required language of the articles was English or Persian. Furthermore, an extensive manual search was conducted on the reference lists of potential articles to uncover further studies.

### 2.2 Eligibility criteria

Included in the meta-analysis were the animal studies examining the impact of saffron and its active components on the intervention group (OVA and intervention) *versus* the OVA-induced asthma group (control group). The established criteria for inclusion to prevent bias were: 1- Sensitization induced by ovalbumin via intraperitoneal injection and nebulizer challenge; 2- The treatment group received either saffron or its key elements in a single treatment for each dose provided. The control group received interventions in the form of non-functional fluid like normal saline without any additional treatment; 3- The primary outcome of investigating the effect of saffron or its active constituents on inflammatory markers such as changes in cytokines and airway inflammatory cells. Investigating the effects of saffron and its active constituents on the mechanisms involved in lung damage post-ovalbumin sensitization was a secondary outcome in the evaluated case.

Excluded from consideration were treatments with other saffron compounds or their active components, sensitization models that did not involve ovalbumin, studies lacking control groups, and those with insufficient data.

### 2.3 Data extraction

The following items were extracted separately by two different authors (F.A and S.S) from the included studies: 1- name of the first author, year of publication, and asthma induction model; 2- characteristics of the studied animals such as the number of animals, species, and sex; 3- information about the intervention group, including the type of intervention used (*Crocus Sativius* extract, Crocin, or Safranal), intervention dose, intervention duration, administration method, and control group information, 4- average and standard deviation for each data. When there was a mismatch in the information, the last referee (MR.A) took action. Each intervention dosage was taken into consideration in the investigation due to varying effects depending on the dose. If meta-analysis data is not available or results are only shown graphically, authors were contacted at least three times. If a response is not given, the digital ruler software measures and removes the data from the graphic charts if no information is present.

### 2.4 Quality assessment

The studies methodological quality was assessed through the Collaborative Evidence-Based Complementary and Alternative Medicine approach to meta-analysis and review of animal data in experimental studies (CAMARADES). The quality of studies was assessed using fifteen different items, categorized from low to high according to specific markers.

### 2.5 Risk of bias assessment

The SYRCLE risk of bias tool for animal studies was utilized to evaluate the potential bias. The modified tool examined twelve questions and assigned each study a risk of bias score as either high, medium, or low.

### 2.6 Statistical analysis

The findings were evaluated using the STATA software version 17. In order to estimate the combined effect size for continuous data, both the confidence interval (CI) and either the mean difference (MD) or standard mean difference (SMD) were taken into consideration with a random effects model. The random effect model was employed to report the results due to the heterogeneity in the studies. Heterogeneity assessment utilized the Q-test and I^2^ index, with significance defined as *p* < 0.10 (I^2^ < 25%, no heterogeneity; I^2^ between 25% and 50%, moderate heterogeneity; I^2^ between 50% and 75%, large heterogeneity; and I^2^ > 75%, extreme heterogeneity). Publication bias was analyzed through the use of the funnel plot examination and Eggers regression test.

## 3 Results

### 3.1 Study selection

**FIGURE 1:**
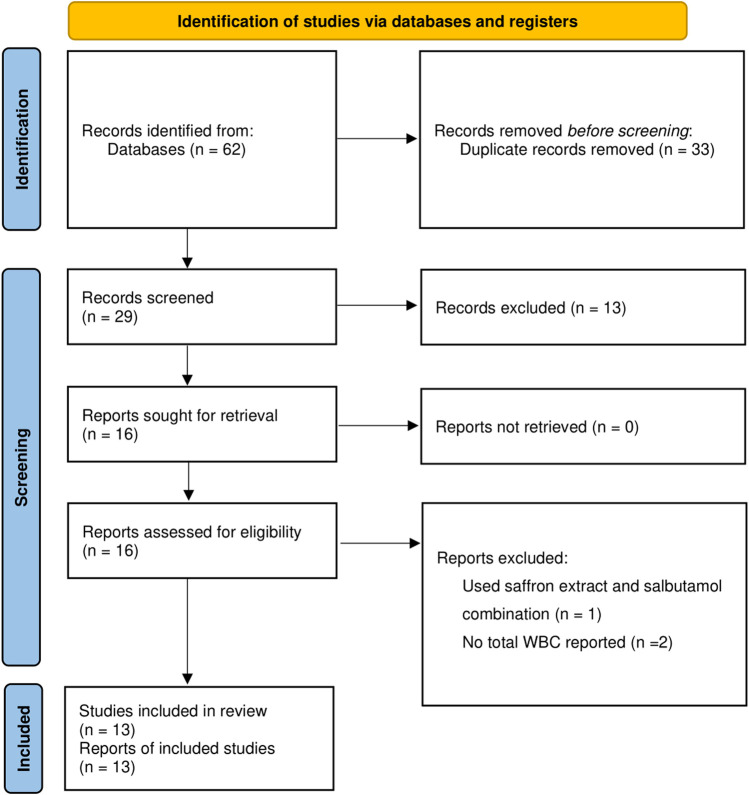
Summary of the process for identifying candidate studies.

By searching the electronic database, a total of 62 records retrieved, and within them, 29 were identified as unique. Thirteen articles did not meet the criteria upon a thorough review of their titles and abstracts; therefore, they were excluded from consideration, and the remaining 16 articles were reviewed according to the inclusion criteria. Three reports were excluded due to a single study looking at saffron and salbutamol together (Nair and Prabhavalkar, 2021), in addition to two studies lacking information on total white blood cell (WBC) (Vosoqi et al., 2013; Ding et al., 2015). Thirteen articles met the criteria for entry into the meta-analysis after a thorough evaluation of their full texts (Bayrami and Boskabady, 2012; Boskabady et al., 2012; Byrami et al., 2013; Gholamnezhad et al., 2013; Mahmoudabady et al., 2013; Vosooghi et al., 2013; Boskabady et al., 2014; Bukhari et al., 2015; Xiong et al., 2015; Yosri et al., 2017; Lertnimitphun et al., 2021; Aslani et al., 2022a; Aslani et al., 2022b) (Figure 1).

### 3.2 Study quality

According to the quality assessment tool with 15 items, all chosen studies received a score higher than ten. All articles were peer reviewed publications. Furthermore, all studies detailed the test methods, intervention dosage, and specific type of intervention employed (*Crocus Sativius* extract, Crocin, or Safranal). However, the topic of conflict of interest was found in 8 studies (Mahmoudabady et al., 2013; Vosooghi et al., 2013; Boskabady et al., 2014; Bukhari et al., 2015; Xiong et al., 2015; Yosri et al., 2017; Aslani et al., 2022a; Aslani et al., 2022b). On the other hand, no studies have discussed the process of calculating sample size, conducting blinded model induction, and blinding the assessment of outcomes (Supplementary Table S2).

### 3.3 Risk of bias

By applying the SYRCLE risk of bias tool to animal studies, it was identified that 12 studies had a low risk of bias and only one study had a medium risk (Supplementary Table S3).

### 3.4 Study characteristics

Table 1 shows a summary of the characteristics of the included studies. The OVA-induced asthma control group (control group) and intervention group (OVA and intervention) each consisted of 268 animals out of a total of 536 across 13 studies. The studies included all had publication dates in English between 2012 and 2022. The experiments utilized BALB/C mice in five studies (Bukhari et al., 2015; Xiong et al., 2015; Lertnimitphun et al., 2021; Aslani et al., 2022a; Aslani et al., 2022b), albino mice in a single study (Yosri et al., 2017), guinea pigs in five studies (Bayrami and Boskabady, 2012; Boskabady et al., 2012; Byrami et al., 2013; Gholamnezhad et al., 2013; Boskabady et al., 2014), and rats in two studies (Mahmoudabady et al., 2013; Vosooghi et al., 2013). Ten studies used male gender (Bayrami and Boskabady, 2012; Boskabady et al., 2012; Byrami et al., 2013; Gholamnezhad et al., 2013; Mahmoudabady et al., 2013; Vosooghi et al., 2013; Boskabady et al., 2014; Yosri et al., 2017; Aslani et al., 2022a; Aslani et al., 2022b), two studies used female gender (Xiong et al., 2015; Lertnimitphun et al., 2021), and one study did not mention the gender of the animal (Bukhari et al., 2015). In all experiments, ovalbumin injection and challenge were utilized to create an asthma model in animals. Four studies utilized Crocin (Xiong et al., 2015; Yosri et al., 2017; Aslani et al., 2022a; Aslani et al., 2022b), while 3 studies focused on Safranal (Boskabady et al., 2014; Bukhari et al., 2015; Lertnimitphun et al., 2021). In addition, 3 studies exclusively used *Crocus sativus* extract (Byrami et al., 2013; Mahmoudabady et al., 2013; Vosooghi et al., 2013), and another 3 studies incorporated both *C. sativus* and Safranal separately (Bayrami and Boskabady, 2012; Boskabady et al., 2012; Gholamnezhad et al., 2013). The doses used for Crocin were 25 mg/kg (intraperitoneally (I.P.)), 50 mg/kg (I.P.), 100 mg/kg (I.P.), 25 mg/kg (orally), and 100 mg/kg (intragastrically); used for Safranal 4 μg/mL (orally), 8 μg/mL (orally), 16 μg/mL (orally), 1 mg/kg (orally), 10 mg/kg (orally), 200 mg/kg (orally), and 500 mg/kg (orally); used for *C. sativus* 20 mg/kg (orally), 40 mg/kg (orally), 80 mg/kg (orally), 50 mg/kg (I.P.), 100 mg/kg (I.P.), and 200 mg/kg (I.P.).

**TABLE 1:** Main characteristics of the included studies in this meta-analysis.

S/N	Study (year)	Species (sex, n = OVA group/intervention group)	Saffron or ingredients	dose and route of administration	Duration of study	Factors for analysis (type of sample)	Intergroup differences (OVA vs. intervention)
1	Aslani (2021) a	BALB/C mice (male, 5/5)	crocin	25 mg/kg, IP	32	WBC (BALF) Neutrophil (BALF) Eosinophil (BALF) Lymphocyte (BALF) Monocyte (BALF) IgE (lung tissue) ATF4 lung tissue) ATF6 lung tissue) GRP78 lung tissue) CHOP lung tissue) XBP1 lung tissue) Caspase 12 lung tissue)	<0.001 NS <0.001 <0.001 <0.001 NS <0.05 NS <0.001 NS NS NS
	Aslani (2021) b	BALB/C mice (male, 5/5)	crocin	50 mg/kg, IP	32	WBC (BALF) Neutrophil (BALF) Eosinophil (BALF) Lymphocyte (BALF) Monocyte (BALF) IgE (lung tissue) ATF4 lung tissue) ATF6 lung tissue) GRP78 lung tissue) CHOP lung tissue) XBP1 lung tissue) Caspase 12 lung tissue)	<0.001 <0.001 <0.001 <0.001 <0.001 NS <0.001 NS <0.001 NS NS <0.05
	Aslani (2021) c	BALB/C mice (male, 5/5)	crocin	100 mg/kg, IP	32	WBC (BALF) Neutrophil (BALF) Eosinophil (BALF) Lymphocyte (BALF) Monocyte (BALF) IgE (lung tissue) ATF4 lung tissue) ATF6 lung tissue) GRP78 lung tissue) CHOP lung tissue) XBP1 lung tissue) Caspase 12 lung tissue)	<0.001 <0.001 <0.001 <0.001 <0.001 <0.01 <0.001 <0.05 <0.001 <0.05 <0.05 <0.05
2	Aslani (2022) a	BALB/C mice (male, 6/6)	crocin	25 mg/kg, IP	32	WBC (BALF) Neutrophil (BALF) Eosinophil (BALF) Lymphocyte (BALF) Monocyte (BALF) IgE (lung tissue) GATA3 (lung tissue) T-bet (lung tissue) miR-106a (lung tissue) miR-146a (lung tissue)	<0.001 <0.05 <0.001 <0.001 <0.001 NS NS NS NS NS
	Aslani (2022) b	BALB/C mice (male, 6/6)	crocin	50 mg/kg, IP	32	WBC (BALF) Neutrophil (BALF) Eosinophil (BALF) Lymphocyte (BALF) Monocyte (BALF) IgE (lung tissue) GATA3 (lung tissue) T-bet (lung tissue) miR-106a (lung tissue) miR-146a (lung tissue)	<0.001 <0.001 <0.001 <0.001 <0.001 NS NS NS <0.01 <0.05
	Aslani (2022) c	BALB/C mice (male, 6/6)	crocin	100 mg/kg, IP	32	WBC (BALF) Neutrophil (BALF) Eosinophil (BALF) Lymphocyte (BALF) Monocyte (BALF) IgE (lung tissue) GATA3 (lung tissue) T-bet (lung tissue) miR-106a (lung tissue) miR-146a (lung tissue)	<0.001 <0.001 <0.001 <0.001 <0.001 <0.01 <0.001 <0.001 <0.001 <0.001
3	Boskabady (2012) a	Guinea pigs (male, 6/6)	*Crocus sativus*	20 mg/kg/orally	32	WBC (BALF) Neutrophil (BALF) Eosinophil (BALF) Lymphocyte (BALF) Monocyte (BALF) Histamine (serum)	<0.01 <0.01 <0.01 NS NS <0.05
	Boskabady (2012) b	Guinea pigs (male, 6/6)	*Crocus sativus*	40 mg/kg/orally	32	WBC (BALF) Neutrophil (BALF) Eosinophil (BALF) Lymphocyte (BALF) Monocyte (BALF) Histamine (serum)	NS NS <0.05 NS <0.05 <0.01
	Boskabady (2012) c	Guinea pigs (male, 6/6)	*Crocus sativus*	80 mg/kg/orally	32	WBC (BALF) Neutrophil (BALF) Eosinophil (BALF) Lymphocyte (BALF) Monocyte (BALF) Histamine (serum)	NS <0.05 NS NS NS <0.001
	Boskabady (2012) a	Guinea pigs (male, 6/6)	Safranal	4 μg/mL/orally	32	WBC (BALF) Neutrophil (BALF) Eosinophil (BALF) Lymphocyte (BALF) Monocyte (BALF) Histamine (serum)	<0.001 <0.05 <0.05 <0.01 <0.01 <0.001
	Boskabady (2012) b	Guinea pigs (male, 6/6)	Safranal	8 μg/mL/orally	32	WBC (BALF) Neutrophil (BALF) Eosinophil (BALF) Lymphocyte (BALF) Monocyte (BALF) Histamine (serum)	NS <NS NS NS NS <0.001
	Boskabady (2012) c	Guinea pigs (male, 6/6)	Safranal	16 μg/mL/orally	32	WBC (BALF) Neutrophil (BALF) Eosinophil (BALF) Lymphocyte (BALF) Monocyte (BALF) Histamine (serum)	NS NS <0.05 <0.01 <0.05 <0.01
4	Boskabady (2014) a	Guinea pigs (male, 6/6)	Safranal	4 μg/mL/orally	32	EC50 Maximum response IL-4 (serum) IFN-γ (serum) NO (serum) Nitrite (serum)	<0.001 NS <0.001 <0.001 <0.001 <0.01
	Boskabady (2014) b	Guinea pigs (male, 6/6)	Safranal	8 μg/mL/orally	32	EC50 Maximum response IL-4 (serum) IFN-γ (serum) NO (serum) Nitrite (serum)	<0.001 NS <0.001 <0.001 <0.01 <0.05
	Boskabady (2014) c	Guinea pigs (male, 6/6)	Safranal	16 μg/mL/orally	32	EC50 Maximum response IL-4 (serum) IFN-γ (serum) NO (serum) Nitrite (serum)	<0.001 NS <0.001 <0.01 <0.01 <0.05
5	Bukhari (2015)	BALB/C mice (-/6/6)	Safranal	1 mg/kg/Orally	28	IL-5 (lung tissue) IL-13 (lung tissue)	NS <0.05
	Bukhari (2015)	BALB/C mice (-/6/6)	Safranal	10 mg/kg/Orally	28	IL-5 (lung tissue) IL-13 (lung tissue)	NS <0.05
6	Bayrami (2012) a	Guinea pigs (male, 6/6)	*Crocus sativus*	20 mg/kg/orally	32	WBC (BALF) Neutrophil (BALF) Eosinophil (BALF) Lymphocyte (BALF) Monocyte (BALF)	NS <0.01 <0.001 NS <0.05
	Bayrami (2012) b	Guinea pigs (male, 6/6)	*Crocus sativus*	40 mg/kg/orally	32	WBC (BALF) Neutrophil (BALF) Eosinophil (BALF) Lymphocyte (BALF) Monocyte (BALF)	NS <0.05 <0.01 NS <0.01
	Bayrami (2012) c	Guinea pigs (male, 6/6)	*Crocus sativus*	80 mg/kg/orally	32	WBC (BALF) Neutrophil (BALF) Eosinophil (BALF) Lymphocyte (BALF) Monocyte (BALF)	<0.05 <0.01 <0.05 <0.05 <0.01
	Bayrami (2012) a	Guinea pigs (male, 6/6)	Safranal	4 μg/mL/orally	32	WBC (BALF) Neutrophil (BALF) Eosinophil (BALF) Lymphocyte (BALF) Monocyte (BALF)	NS <0.001 <0.001 NS <0.01
	Bayrami (2012) b	Guinea pigs (male, 6/6)	Safranal	8 μg/mL/orally	32	WBC (BALF) Neutrophil (BALF) Eosinophil (BALF) Lymphocyte (BALF) Monocyte (BALF)	NS <0.01 <0.001 <0.05 <0.01
	Bayrami (2012) c	Guinea pigs (male, 6/6)	Safranal	16 μg/mL/orally	32	WBC (BALF) Neutrophil (BALF) Eosinophil (BALF) Lymphocyte (BALF) Monocyte (BALF)	NS <0.05 <0.001 NS <0.001
7	Byrami (2013) a	Guinea pigs (male, 6/6)	*Crocus sativus*	20 mg/kg/orally	32	EC50 Maximum response IL-4 (serum) IFN-γ (serum) NO (serum) Nitrite (serum)	<0.001 <0.05 <0.001 NS <0.05 <0.05
	Byrami (2013) b	Guinea pigs (male, 6/6)	*Crocus sativus*	40 mg/kg/orally	32	EC50 Maximum response IL-4 (serum) IFN-γ (serum) NO (serum) Nitrite (serum)	<0.05 <0.001 <0.001 <0.01 <0.01 <0.01
	Byrami (2013) c	Guinea pigs (male, 6/6)	*Crocus sativus*	80 mg/kg/orally	32	EC50 Maximum response IL-4 (serum) IFN-γ (serum) NO (serum) Nitrite (serum)	<0.05 <0.05 <0.001 <0.001 <0.001 <0.01
8	Gholamnezhad (2013) a	Guinea pigs (male, 6/6)	*Crocus sativus*	20 mg/kg/orally	32	Endothelin (serum)	NS
	Gholamnezhad (2013) b	Guinea pigs (male, 6/6)	*Crocus sativus*	40 mg/kg/orally	32	Endothelin (serum)	<0.001
	Gholamnezhad (2013) c	Guinea pigs (male, 6/6)	*Crocus sativus*	80 mg/kg/orally	32	Endothelin (serum)	<0.001
	Gholamnezhad (2013) a	Guinea pigs (male, 6/6)	Safranal	4 μg/mL/orally	32	Endothelin (serum)	<0.001
	Gholamnezhad (2013) b	Guinea pigs (male, 6/6)	Safranal	8 μg/mL/orally	32	Endothelin (serum)	<0.01
	Gholamnezhad (2013) c	Guinea pigs (Male, 6/6)	Safranal	16 μg/mL/orally	32	Endothelin (serum)	NS
9	Lertnimitphun (2021) a	BALB/C mice (Female, 10/10)	Safranal	200 mg/kg/orally	25	IgE (serum) IL-4 (lung tissue) IL-5 (lung tissue) IL-13 (lung tissue) IFN-γ (lung tissue)	NS <0.001 <0.001 <0.001 NS
	Lertnimitphun (2021) b	BALB/C mice (Female, 10/10)	Safranal	500 mg/kg/orally	25	IgE (serum) IL-4 (lung tissue) IL-5 (lung tissue) IL-13 (lung tissue)) IFN-γ (lung tissue)	<0.05 <0.001 <0.001 <0.001 NS
10	Mahmoudabady (2013) a	Rat (male, 8/8)	*Crocus sativus*	50 mg/kg/IP	32	WBC (BALF) Neutrophil (BALF) Eosinophil (BALF) Lymphocyte (BALF)	<0.001 NS NS <0.05
	Mahmoudabady (2013) b	Rat (male, 8/8)	*Crocus sativus*	100 mg/kg/IP	32	WBC (BALF) Neutrophil (BALF) Eosinophil (BALF) Lymphocyte (BALF)	<0.001 <0.01 <0.001 NS
	Mahmoudabady (2013) c	Rat (male, 8/8)	*Crocus sativus*	200 mg/kg/IP	32	WBC (BALF) Neutrophil (BALF) Eosinophil (BALF) Lymphocyte (BALF)	<0.001 NS <0.001 NS
11	Vosoghi (2013) a	Rat (male, 8/8)	*Crocus sativus*	50 mg/kg/IP	32	WBC (BALF) Neutrophil (BALF) Eosinophil (BALF) Lymphocyte (BALF)	<0.01 NS <0.001 NS
	Vosoghi (2013) b	Rat (male, 8/8)	*Crocus sativus*	100 mg/kg/IP	32	WBC (BALF) Neutrophil (BALF) Eosinophil (BALF) Lymphocyte (BALF)	<0.01 NS <0.001 <0.05
	Vosoghi (2013) c	Rat (male, 8/8)	*Crocus sativus*	200 mg/kg/IP	32	WBC (BALF) Neutrophil (BALF) Eosinophil (BALF) Lymphocyte (BALF)	NS NS <0.001 NS
12	Xiong (2015)	BALB/C mice (Female, 10/10)	Crocin	100 mg/kg/intragastrically	32	WBC (BALF) Neutrophil (BALF) Eosinophil (BALF) Lymphocyte (BALF) Monocyte (BALF) IgE (BALF) IL-4 (BALF) IL-5 (BALF) IL-10 (BALF) IL-13 (BALF)	<0.05 <0.01 <0.01 <0.01 <0.05 <0.01 <0.01 <0.01 NS <0.01
13	Yosri (2017)	Albino mice (male, 10/10)	Crocin	25 mg/kg/orally	16	WBC (BALF) Neutrophil (BALF) Eosinophil (BALF) Monocyte (BALF) IL-4 (lung tissue) IL-13 (lung tissue) TNF-α (lung tissue) MDA (lung tissue) SOD (lung tissue) GSH (lung tissue) LDH (serum) Catalase (serum)	NS <0.05 <0.05 <0.05 <0.05 <0.05 <0.05 <0.05 <0.05 <0.05 <0.05 <0.05

ATF4: activating transcription factor 4, ATF6: activating transcription factor 6, BALF: bronchoalveolar lavage fluid, CHOP: C/EBP, homologous protein, EC50: half maximal effective concentration, GATA3: GATA, Binding Protein 3, GRP78: glucose regulatory protein 78, GSH: glutathione, IFN-γ: interferon‐gamma, IgE: immunoglobulin E, I.P.: intraperitoneally, LDH: lactate dehydrogenase, IL: interleukin, MDA: malondialdehyde, miR: microRNA, NO: nitric oxide, NS: non-significant, SOD: superoxide dismutase, T-bet: T-box transcription factor, WBC: white blood cell, XBP1: transcription factor X-box binding protein.

The total WBC, neutrophil, and eosinophil counts were reported in eight studies (Bayrami and Boskabady, 2012; Boskabady et al., 2012; Mahmoudabady et al., 2013; Vosooghi et al., 2013; Xiong et al., 2015; Yosri et al., 2017; Aslani et al., 2022a; Aslani et al., 2022b), lymphocyte count in 7 (Bayrami and Boskabady, 2012; Boskabady et al., 2012; Mahmoudabady et al., 2013; Vosooghi et al., 2013; Xiong et al., 2015; Aslani et al., 2022a; Aslani et al., 2022b), and monocyte count in 7 studies (Bayrami and Boskabady, 2012; Boskabady et al., 2012; Mahmoudabady et al., 2013; Xiong et al., 2015; Yosri et al., 2017; Aslani et al., 2022a; Aslani et al., 2022b) from BALF samples. In total, four studies documented immunoglobulin E (IgE) values (Xiong et al., 2015; Lertnimitphun et al., 2021; Aslani et al., 2022a; Aslani et al., 2022b); among them, two utilized lung tissue samples, one relied on BALF samples, and another used serum samples. Five research papers detailed IL-4 levels (Byrami et al., 2013; Boskabady et al., 2014; Xiong et al., 2015; Yosri et al., 2017; Lertnimitphun et al., 2021), with three utilizing serum samples, one utilizing lung tissue samples, and one utilizing BALF samples. IL-5 levels were reported in three studies (Bukhari et al., 2015; Xiong et al., 2015; Lertnimitphun et al., 2021), with two cases utilizing lung tissue samples and one case using BALF samples. Interferon‐gamma (IFN-γ) levels were measured in three studies (Byrami et al., 2013; Boskabady et al., 2014; Lertnimitphun et al., 2021), with two utilizing serum samples and one focusing on lung tissue samples. Four investigations detailed IL-13 levels (Bukhari et al., 2015; Xiong et al., 2015; Yosri et al., 2017; Lertnimitphun et al., 2021), with three utilizing lung tissue samples and one using BALF samples. Two investigations detailed the serum nitric oxide (NO) concentrations (Byrami et al., 2013; Boskabady et al., 2014). One study reported serum histamine levels (Boskabady et al., 2012). In two studies, levels of serum nitrite were noted (Byrami et al., 2013; Boskabady et al., 2014). Two investigations documented half maximal effective concentration (EC50) and maximum response measurements (Byrami et al., 2013; Boskabady et al., 2014). Levels of serum endothelin were reported in a study results (Gholamnezhad et al., 2013). One study reported the levels of tumor necrosis factor alpha (TNF-α), malondialdehyde (MDA), superoxide dismutase (SOD), and glutathione (GSH) in lung tissue, as well as the levels of serum lactate dehydrogenase (LDH) and catalase (Yosri et al., 2017). One study reported the levels of IL-10 in BALF sample (Xiong et al., 2015). One study reported the levels of T-box transcription factor (T-bet), GATA Binding Protein 3 (GATA3), microRNA (miR)-146a, and miR-106a in lung tissue samples (Aslani et al., 2022b). Finally, a study reported the levels of activating transcription factor 4 (ATF4), activating transcription factor 6 (ATF6), glucose regulatory protein 78 (GRP78), C/EBP homologous protein (CHOP), transcription factor X-box binding protein (XBP1), and caspase 12 in lung tissue samples (Aslani et al., 2022a).

### 3.5 Effectiveness

#### 3.5.1 Effect of saffron and its ingredients on BALF total WBC count

**FIGURE 2:**
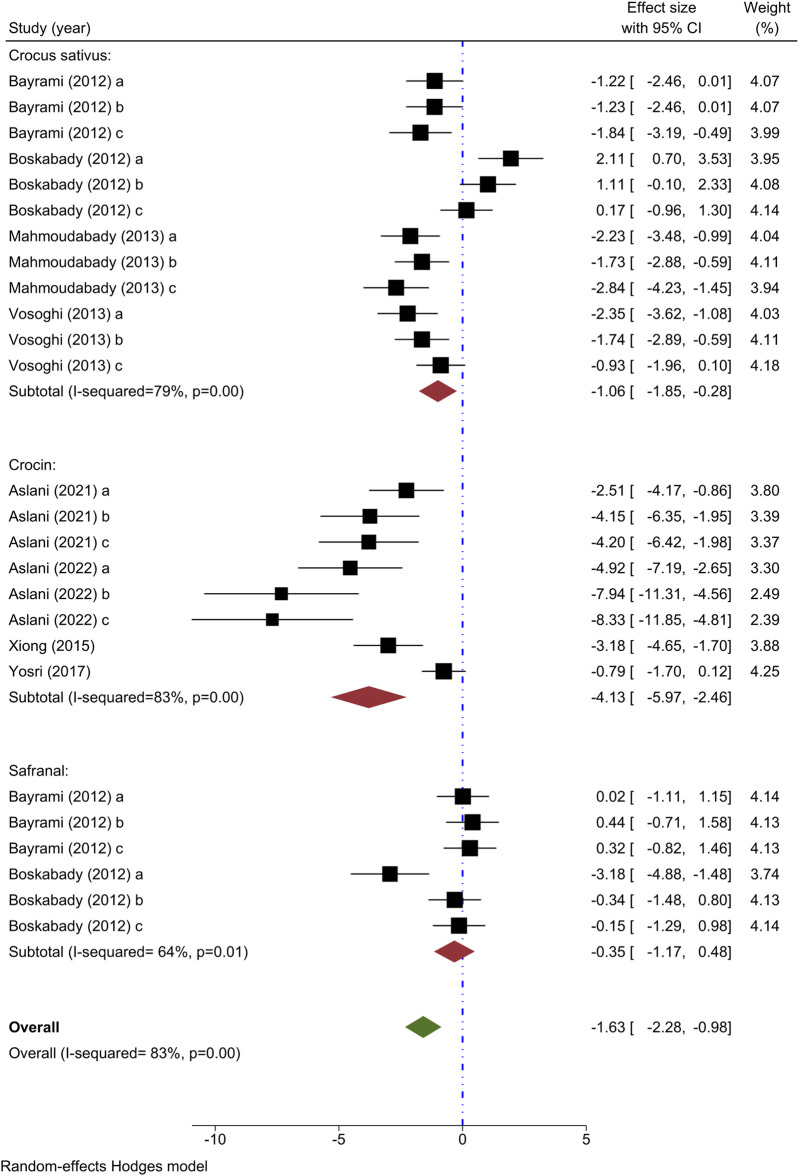
Forest plot detailing standardized mean differences (SMD) and 95% confidence intervals (CIs) in the studies reporting the effect of Crocus sativus, Crocin, and Safranal on total WBC count in intervention groups compared to OVA-induced asthma group.

Total WBC count has been utilized as an outcome measure in eight investigations. Four investigations have explored the impact of various levels of *C. sativus* on total WBC counts (Bayrami and Boskabady, 2012; Boskabady et al., 2012; Mahmoudabady et al., 2013; Vosooghi et al., 2013), with all studies demonstrating a decrease in levels except for one (Boskabady et al., 2012). A meta-analysis of four studies indicated a notable impact of *C. sativus* in decreasing the total WBC count in comparison to the asthma model group [n = 152, SMD = −1.06, 95% CI (−1.85 to −0.28), P< 0.01] (Figure 2).

The impact of crocin on total WBC count was studied in four different experiments (Xiong et al., 2015; Yosri et al., 2017; Aslani et al., 2022a; Aslani et al., 2022b), each demonstrating a decrease when compared to the control group. A meta-analysis incorporating findings from 4 studies indicated a substantial decrease in total WBC value by crocin in comparison to the asthma model group [n = 106, SMD = −4.13, 95% CI (−5.97 to −2.46), P< 0.001] (Figure 2).

Two studies examined the impact of varying concentrations of Safranal on total WBC count (Bayrami and Boskabady, 2012; Boskabady et al., 2012), finding a notable increase in total WBC count within the intervention group compared to those with asthma. When examining two studies through meta-analysis, it was found that Safranal did not result in any significant change in the total WBC count when compared to the asthma model group [n = 72, SMD = −0.35, 95% CI (−1.17 to 0.48)] (Figure 2).

Overall, the meta-analysis confirmed the ability of *C. sativus*, Crocin, and Safranal to reduce total WBC counts when compared to the asthma model group [n = 328, SMD = −1.63, 95% CI (−2.28 to −0.98), P< 0.001] (Figure 2).

#### 3.5.2 Effect of saffron and its ingredients on BALF eosinophil count

**FIGURE 3:**
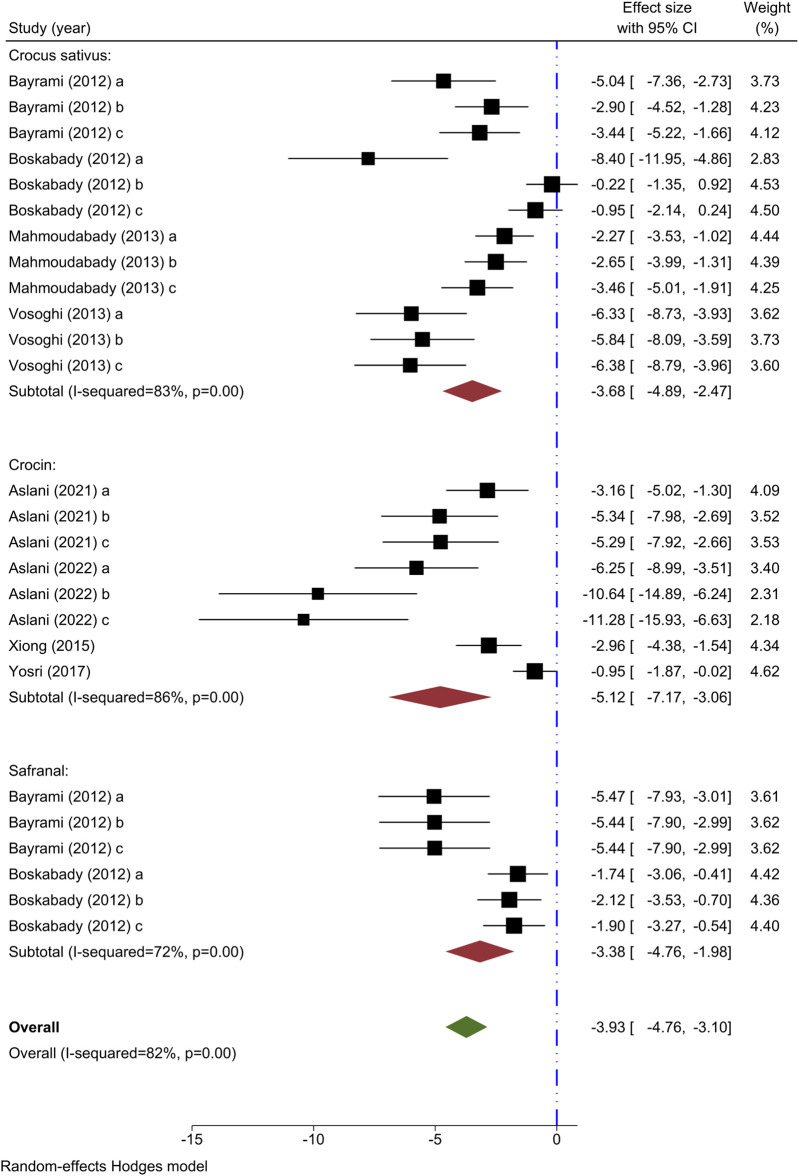
Forest plot detailing standardized mean differences (SMD) and 95% confidence intervals (CIs) in the studies reporting the effect of Crocus sativus, Crocin, and Safranal on eosinophil count in intervention groups compared to OVA-induced asthma group.

The outcome measure in eight experiments was the eosinophil count. Decreasing effects on eosinophil counts were observed in all four studies analyzing the impact of different levels of *C. sativus* (Bayrami and Boskabady, 2012; Boskabady et al., 2012; Mahmoudabady et al., 2013; Vosooghi et al., 2013), compared to the model group. A meta-analysis pooling data from 4 studies demonstrated a noteworthy impact of *C. sativus* in lowering eosinophil levels compared to the asthma control group [n = 152, SMD = −3.68, 95% CI (−4.89 to −2.47), *P* < 0.001] (Figure 3).

Four experiments have examined the impact of varying levels of crocin on eosinophil count (Xiong et al., 2015; Yosri et al., 2017; Aslani et al., 2022a; Aslani et al., 2022b), each demonstrating a decrease in comparison to the asthma group. Crocin was found to significantly lower eosinophil count compared to the asthma model group in a meta-analysis of 4 studies [n = 106, SMD = −5.12, 95% CI (−7.17 to −3.06), *P* < 0.001] (Figure 3).

Two studies examined the impact of varying concentrations of Safranal on eosinophil count (Bayrami and Boskabady, 2012; Boskabady et al., 2012), finding a reduction in the intervention group compared to the asthma group. Safranal significantly decreased eosinophil count in comparison to the asthma group, according to a meta-analysis of two studies [n = 72, SMD = −3.38, 95% CI (−4.76 to −1.98), *P* < 0.001] (Figure 3).

Eosinophil count was shown to decrease when compared to the asthma group in the overall meta-analysis of *C. sativus*, Crocin, and Safranal [n = 328, SMD = −3.93, 95% CI (−4.76 to −3.10), P< 0.001] (Figure 3).

#### 3.5.3 Effect of saffron and its ingredients on BALF neutrophil count

**FIGURE 4:**
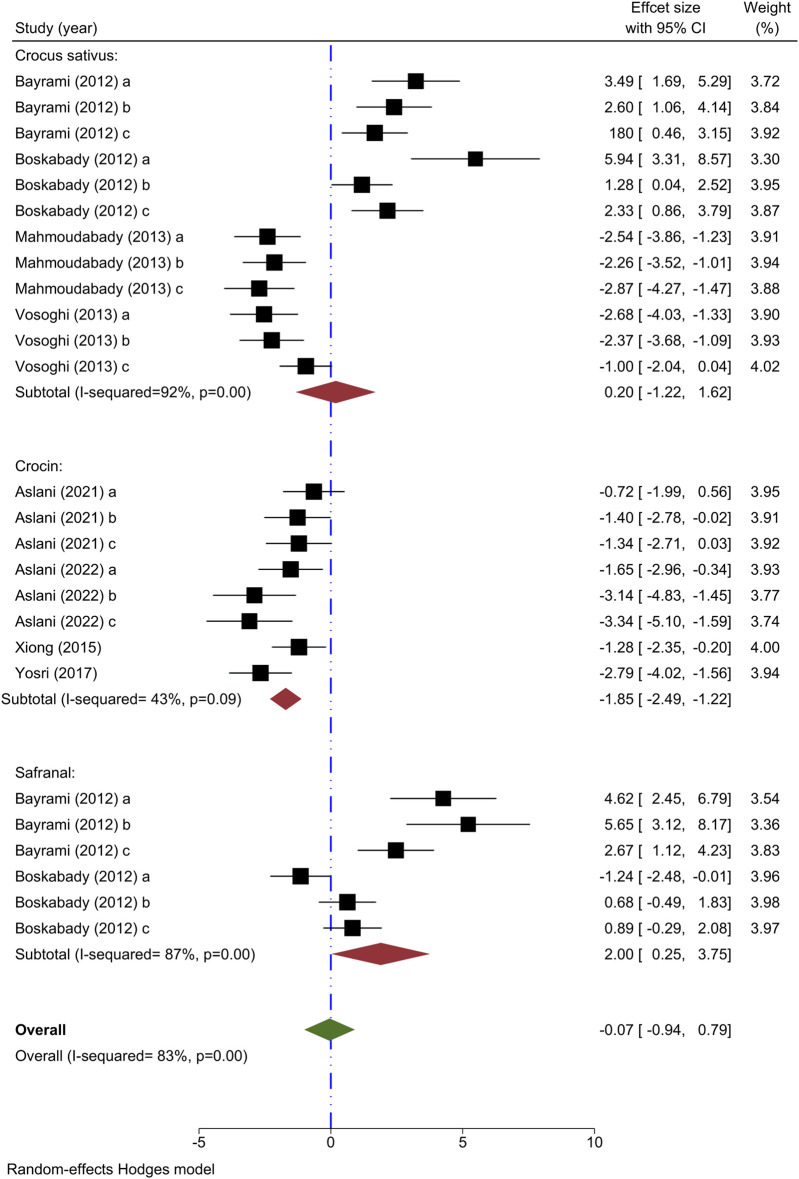
Forest plot detailing standardized mean differences (SMD) and 95% confidence intervals (CIs) in the studies reporting the effect of Crocus sativus, Crocin, and Safranal on neutrophil count in intervention groups compared to OVA-induced asthma group.

Neutrophil count has been utilized as an outcome measure in eight studies. The impact of *C. sativus* at varying concentrations on neutrophil amounts was examined in four studies (Bayrami and Boskabady, 2012; Mahmoudabady et al., 2013; Vosooghi et al., 2013; Boskabady et al., 2014); while two showed decreased levels, the other two demonstrated an increase post-intervention relative to the asthma model group. The analysis of 4 studies indicated that *C. sativus* did not show any notable changes in neutrophil levels compared to the asthma group [n = 152, SMD = 0.20, 95% CI (−1.22–1.62)] (Figure 4).

The effect of varying concentrations of crocin on neutrophil levels was analyzed in 4 different studies (Xiong et al., 2015; Yosri et al., 2017; Aslani et al., 2022a; Aslani et al., 2022b), all indicating decreased effects when compared to the asthma group. Meta-analysis of 4 studies showed a significant effect of crocin to reduce the neutrophil amount compared to the asthma model group [n = 106, SMD = −1.85, 95% CI (−2.49 to −1.22), *P* < 0.001] (Figure 4).

Two studies have examined the impact of varying levels of Safranal on neutrophil count (Bayrami and Boskabady, 2012; Boskabady et al., 2012), finding that the intervention group showed a significant increase in neutrophil count compared to the asthma model group. In a meta-analysis of two studies, it was found that Safranal did not have a significant impact on the neutrophil count [n = 72, SMD = 2.00, 95% CI (0.25–3.75)] (Figure 4).

The overall meta-analysis revealed that *C. sativus*, Crocin, and Safranal did not impact the neutrophil levels [n = 328, SMD = −0.07, 95% CI (−0.94 to 0.79)] (Figure 4).

#### 3.5.4 Effect of saffron and its ingredients on BALF lymphocyte count

**FIGURE 5:**
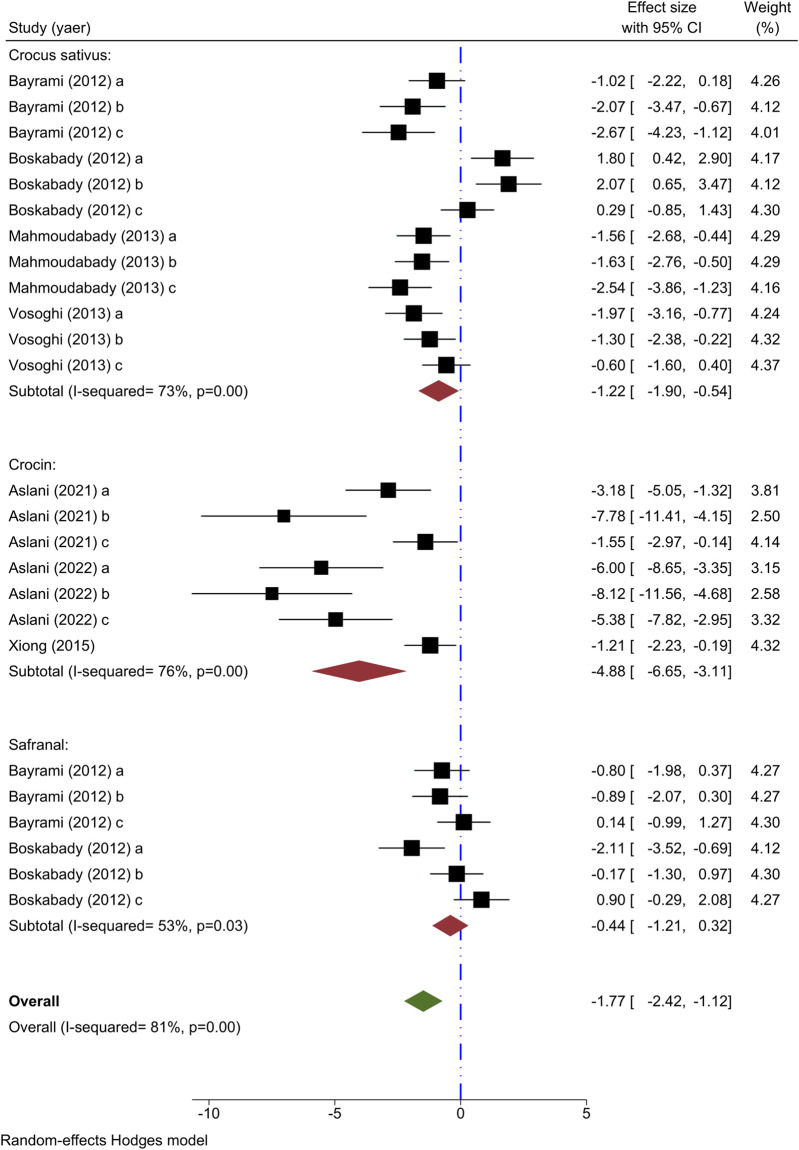
Forest plot detailing standardized mean differences (SMD) and 95% confidence intervals (CIs) in the studies reporting the effect of Crocus sativus, Crocin, and Safranal on lymphocyte count in intervention groups compared to OVA-induced asthma group.

The outcome measure for seven studies involved analyzing lymphocyte count. Four studies delved into the impact of different concentrations of *C. sativus* on lymphocyte count (Bayrami and Boskabady, 2012; Boskabady et al., 2012; Mahmoudabady et al., 2013; Vosooghi et al., 2013), showing a decrease in numbers except for the study by Boskabady et al. The meta-analysis of 4 studies revealed a marked decrease in lymphocyte count when using *C. sativus* [n = 152, SMD = −1.22, 95% CI (−1.90 to −0.54), *P* < 0.001] (Figure 5).

The influence of varying concentrations of crocin on lymphocyte counts was studied in three trials (Xiong et al., 2015; Aslani et al., 2022a; Aslani et al., 2022b), each demonstrating decreased effects relative to the asthma group. The meta-analysis of three studies demonstrated a noticeable impact of crocin in lowering lymphocyte levels [n = 86, SMD = −4.88, 95% CI (−6.65 to −3.11), *P* < 0.001] (Figure 5).

Two experiments were conducted to explore the impact of varying levels of Safranal on lymphocyte count (Bayrami and Boskabady, 2012; Boskabady et al., 2012), with findings indicating a rise in lymphocytes among the intervention group in contrast to the asthma control group. A meta-analysis of two studies showed that Safranal had no significant effect on the lymphocyte count [n = 72, SMD = −0.44, 95% CI (−1.21 to 0.32)] (Figure 5).

*Crocus sativus*, Crocin, and Safranal were found to have diminishing effects on lymphocyte count as shown by the overall meta-analysis [n = 308, SMD = −1.77, 95% CI (−2.42 to −1.12), *P* < 0.001] (Figure 5).

#### 3.5.5 Effect of saffron and its ingredients on BALF monocyte count

**FIGURE 6:**
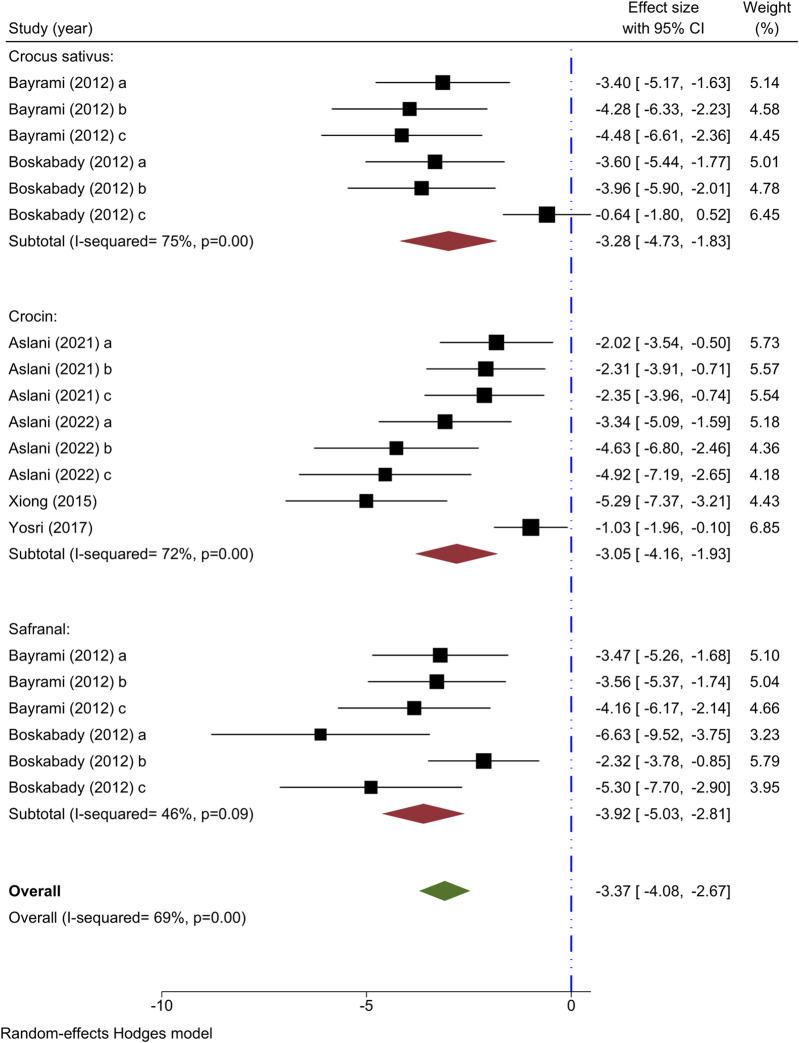
Forest plot detailing standardized mean differences (SMD) and 95% confidence intervals (CIs) in the studies reporting the effect of Crocus sativus, Crocin, and Safranal on monocyte count in intervention groups compared to OVA-induced asthma group.

Six studies have used monocyte count as an outcome measure. Two studies have explored the impact of various levels of *C. sativus* on monocyte count (Bayrami and Boskabady, 2012; Boskabady et al., 2012), all revealing decreased. The results of a meta-analysis involving two studies indicated that *C. sativus* effectively lowered monocyte counts [n = 72, SMD = −3.28, 95% CI (−4.73 to −1.83), *P* < 0.001] (Figure 6).

Four research studies have examined the impact of various concentrations of crocin on monocyte count (Xiong et al., 2015; Yosri et al., 2017; Aslani et al., 2022a; Aslani et al., 2022b), all revealing decreased effects. The meta-analysis of 4 studies indicated a significant reduction in monocyte count with the administration of crocin [n = 106, SMD = −3.05, 95% CI (−4.16 to −1.93), *P* < 0.001] (Figure 6).

The monocyte count was observed to decrease in subjects receiving varying concentrations of Safranal in two studies (Bayrami and Boskabady, 2012; Boskabady et al., 2012). Safranal had a noticeable impact on lowering monocyte levels, as indicated by a meta-analysis of two studies [n = 72, SMD = −3.92, 95% CI (−5.03 to −2.81), *P* < 0.001] (Figure 6).

Through the overall meta-analysis, it was revealed that *C. sativus*, as well as Crocin and Safranal, had a notable impact on reducing monocyte counts [n = 248, SMD = −3.37, 95% CI (−4.08 to −2.67), *P* < 0.001] (Figure 6).

#### 3.5.6 Effect of saffron and its constituents on EC50 and maximum response value

Two studies have used EC50 value as an outcome measure (Byrami et al., 2013; Boskabady et al., 2014). The findings revealed that *C. sativus* and Safranal both had the ability to raise the EC50 value when compared to the asthma model group. A synthesis of two studies demonstrated a significant effect of *C. sativus* and Safranal in enhancing the EC50 value [n = 72, SMD = 3.34, 95% CI (1.80–4.88), *P* < 0.001] (Supplementary Figure S1).

Two studies have used maximum response value as an outcome measure (Byrami et al., 2013; Boskabady et al., 2014). According to the data, *C. sativus* and Safranal were found to have a decreasing effect on the maximum response value. Through meta-analysis of two studies, it was found that *C. sativus* and Safranal had a noteworthy effect in lowering the maximum response value [n = 72, SMD = −1.49, 95% CI (−2.02 to −0.96), *P* < 0.001] (Supplementary Figure S1).

#### 3.5.7 Effect of saffron and its ingredients on IL-4, IL-5, IL-13, and IFN-γ levels

IL-4 levels have been utilized as an outcome measure in four different studies. The study findings indicated that *C. sativus* (Byrami et al., 2013), Crocin (Xiong et al., 2015; Yosri et al., 2017), and Safranal (Boskabady et al., 2014; Lertnimitphun et al., 2021) were effective in lowering IL-4 levels. The synthesis of data from 5 studies indicated a marked decrease in IL-4 levels with the use of *C. sativus*, Crocin, and Safranal [n = 152, SMD = −4.77, 95% CI (−6.30 to −3.24), *P* < 0.001] (Supplementary Figure S2).

Three studies have used IL-5 levels as an outcome measure. According to the findings, Crocin (Xiong et al., 2015) and Safranal (Bukhari et al., 2015; Lertnimitphun et al., 2021) were found to lower IL-5 levels. A significant reduction in IL-5 levels was found in a meta-analysis of 3 studies examining the effects of Crocin and Safranal [n = 84, SMD = −3.36, 95% CI (−5.40 to −1.33), *P* < 0.01] (Supplementary Figure S2).

The outcome measure of four studies involved IL-13 levels. A decrease in IL-13 levels was observed with the use of Crocin (Xiong et al., 2015; Yosri et al., 2017) and Safranal (Bukhari et al., 2015; Lertnimitphun et al., 2021) as indicated by the results. The results of analyzing 4 studies demonstrated a substantial decrease in IL-13 levels with the use of Crocin and Safranal [n = 104, SMD = −2.11, 95% CI (−3.01 to −1.21), *P* < 0.001] (Supplementary Figure S2).

The outcome measure in three studies involved examining IFN-γ levels. The findings indicated that *C. sativus* (Byrami et al., 2013; Boskabady et al., 2014) and Safranal (Lertnimitphun et al., 2021) were associated with raised IFN-γ levels. A meta-analysis of three studies demonstrated a significant impact of *C. sativus* and Safranal on elevating IFN-γ levels [n = 84, SMD = 2.40, 95% CI (1.23–3.57), *P* < 0.001] (Supplementary Figure S2).

#### 3.5.8 Effect of saffron and its ingredients on IgE, histamine, NO, and nitrite levels

As an outcome measure, IgE levels were employed in four studies. Crocin (Xiong et al., 2015; Aslani et al., 2022a; Aslani et al., 2022b) and Safranal (Lertnimitphun et al., 2021) were shown to have a positive impact on reducing IgE levels. The meta-analysis confirmed the effectiveness of Crocin and Safranal in lowering IgE levels based on data from 4 studies [n = 126, SMD = −1.30, 95% CI (−1.87 to −0.73), *P* < 0.001] (Supplementary Figure S3).

In one study, histamine levels served as the outcome measure. In the study, it was found that *C. sativus* (Boskabady et al., 2012) and Safranal (Boskabady et al., 2012) were effective in lowering histamine levels. An analysis pooling data from one study indicated a marked reduction in histamine levels with the use of *C. sativus* and Safranal [n = 72, SMD = −3.86, 95% CI (−5.80 to −1.92), *P* < 0.001] (Supplementary Figure S3).

NO levels served as the outcome measure in two distinct studies. According to the results, *C. sativus* (Byrami et al., 2013) and Safranal (Boskabady et al., 2014) demonstrated the ability to decrease NO levels. Through a meta-analysis of two studies, it was determined that *C. sativus* and Safranal are effective in decreasing NO levels [n = 72, SMD = −2.69, 95% CI (−3.72 to −1.66), *P* < 0.001] (Supplementary Figure S3).

In two studies, nitrite levels were chosen as an outcome indicator. The study demonstrated that *C. sativus* (Byrami et al., 2013) and Safranal (Boskabady et al., 2014) were effective in decreasing nitrite levels. Meta-analysis of two studies showed a significant effect of *C. sativus* and Safranal to reduce the nitrite level [n = 72, SMD = −2.22, 95% CI (−2.85 to −1.58), *P* < 0.001] (Supplementary Figure S3).

#### 3.5.9 Effect of saffron and its ingredients on ER stress markers

One study have used ER stress markers value (ATF4, ATF6, CHOP, GRP78, XBP1, and Caspase 12) as an outcome measure (Aslani et al., 2022a). Except for ATF6, crocin significantly decreased levels of ATF4, CHOP, GRP78, XBP1, and Caspase 12 (Supplementary Figure S4).

#### 3.5.10 Effect of saffron and its ingredients on other signaling pathways

One study have used GATA3, T-bet, miR-106a, and miR-146a as an outcome measure (Aslani et al., 2022b). The study concluded that crocin effectively decreased levels of GATA3, miR-106a, and miR-146a. In contrast, crocin markedly raises T-bet levels. (Supplementary Figure S5).

Endothelin levels were utilized as an outcome measure in a study. The findings indicated that *C. sativus* (Gholamnezhad et al., 2013) and Safranal (Gholamnezhad et al., 2013) had a marked decrease on endothelin levels (Supplementary Figure S5).

In one experiment, TNF-α, MDA, and LDH were employed as outcome for evaluation (Yosri et al., 2017). Crocin was found to have a significant effect in decreasing TNF-α, MDA, and LDH levels. On the other hand, one study used GSH, SOD, catalase (Yosri et al., 2017), and IL-10 (Xiong et al., 2015) as an outcome measure. Significantly elevated levels of GSH, SOD, catalase, and IL-10 were observed with the use of crocin in the study.

### 3.6 Sensitivity analysis

A sensitivity analysis was performed by removing data from the model to examine the impact of high risk of bias and low quality studies on the robustness of the results of the current study. The results revealed an overall effect size in relation to total WBC [SMD = −1.63, 95% CI (−2.28 to −0.98); I^2^: 83%, *P* < 0.001], eosinophil count [SMD = −3.93, 95% CI (−4.76 to −3.10); I^2^: 82%, *P* < 0.001], neutrophil count [SMD = −0.07, 95% CI (−0.94 to 0.79); I^2^: 89%, *P* < 0.001], lymphocyte count [SMD = −1.77, 95% CI (−2.42 to −1.12); I^2^: 81%, *P* < 0.001], monocyte count [SMD = −3.37, 95% CI (−4.08 to −2.67); I^2^: 69%, *P* < 0.001], IFN-γ level [SMD = 2.40, 95% CI (1.23–3.57); I^2^: 81%, *P* < 0.001], IL-4 level [SMD = −4.77, 95% CI (−6.30 to −3.24); I^2^: 86%, *P* < 0.001], IL-5 level [SMD = −3.63, 95% CI (−5.40 to −3.24); I^2^: 89%, *P* < 0.001], IL-13 level [SMD = −2.11, 95% CI (−3.01 to −1.21); I^2^: 68%, *P* < 0.01], and IgE level [SMD = −1.30, 95% CI (−1.87 to −0.73); I^2^: 45%, *P* < 0.05].

### 3.7 Publication bias

Based on the analysis of the funnel plots, it was revealed that the studies included in the data analysis had publication bias (Supplementary Figure S6A–J). It also confirmed the publication bias based on the findings of Egger’s linear regression test significantly regarding the data related to total WBC (*p* = 0.000), eosinophil count (*p* = 0.000), neutrophil count (*p* = 0.005), lymphocyte count (*p* = 0.000), monocyte count (*p* = 0.000), IL-4 level (*p* = 0.000), and IL-5 level (*p* = 0.010). However, the eggers test for the IgE (*p* = 0.070), IL-13 (*p* = 0.16), and IFN-γ (*p* = 0.000) levels indicated there is no significant publication bias.

## 4 Discussion

The key finding from the current preclinical systematic investigation on the efficacy of saffron and its constituents in the ovalbumin-induced asthma model were: 1- the protective effects of saffron and its constituents were evidenced through their ability to decrease inflammatory pathways, including the modulation of airway inflammation, cytokines, and inflammatory mediators (IL-4, IL-5, IL-13, IFN-γ, histamine, endothelin, nitrite and NO), 2- the use of saffron and its components resulted in enhanced respiratory function through decreased responsiveness of the airways to methacoline (through improve of EC50 and maximum response values), and 3- research conducted on the efficacy of saffron and its components found that the regulation of ER stress pathways, modulation of miRNA expression, and adjustment of the GATA3/T-bet ratio played key roles in these mechanisms.

While there have been numerous studies on saffron and its compounds in various disorders, their underlying biological mechanisms are still largely unknown. Asthma is an inflammatory disease of the airways that activates the T-helper two immune response (Durrant and Metzger, 2010). There is numerous evidence indicating a growth in the overall number of WBCs and differential cells within the respiratory systems of asthmatic patients (Aslani et al., 2017; Solomon et al., 2022). Pathological assessment has revealed the infiltration of inflammatory cells into the airways in the ovalbumin-induced asthma model (Saadat et al., 2015; Ahmadi et al., 2016). In BALF samples, there is a marked increase in eosinophils, lymphocytes, and neutrophils, particularly in severe asthma (Aslani et al., 2017). One potential mechanism by which saffron and its ingredients protect against asthma is through their ability to reduce airway inflammation cells in the ovalbumin-induced model. In addition, asthma conditions have been linked to increased levels of inflammatory cytokines such as IL-4, IL-5, IL-17, IL-13, and TNF-α in addition to decreased IFN-γ and IL-10 (Hammad and Lambrecht, 2021). Saffron effectiveness in animal studies may be attributed to its ability to lower levels of inflammatory cytokines. Studies have shown a correlation between the elevated in inflammatory cells and cytokines, as well as the development of AHR and changes in airway remodeling (Savin et al., 2023). The ability of saffron to lower AHR in animal studies has been established, but the precise process by which this occurs is still unclear; it is believed that a decrease in inflammatory elements could be a contributing factor.

The ovalbumin-induced asthma model has demonstrated elevated levels of inflammatory mediators, including NO (Byrami et al., 2013; Boskabady et al., 2014), endothelin (Gholamnezhad et al., 2013), histamine (Boskabady et al., 2012), LDH (Yosri et al., 2017), and nitrite (Byrami et al., 2013; Boskabady et al., 2014). The bronchoconstrictor effect of endothelin has been well-reported in animal and human studies. The role of endothelin in asthma has been demonstrated through its effects on arachidonic acid metabolites, histamine, and leukotrienes, with evidence suggesting direct action on airway smooth muscle in human studies (Chalmers et al., 1997). NO and nitrite play a dual role in asthma by exerting both positive and negative impacts. While NO production through cyclic GMP regulation promotes bronchodilation, excessive levels can result in elevated mucus secretion (Rihák et al., 2010; Prado et al., 2011). Studies have demonstrated that when inducible nitric oxide synthases (iNOS) produces high levels of NO, the lung experiences an influx of inflammatory cells such as eosinophils and lymphocytes. Moreover, NO and nitrite are pivotal in triggering cellular damage and AHR by activating the oxidative stress pathway (Rihák et al., 2010; Prado et al., 2011). Another mechanism in which saffron may impact ovalbumin-induced asthma is by controlling the inflammatory mediators. The use of saffron resulted in a notable decrease in endothelin, NO, nitrite, LDH, and histamine levels, with the findings from the present meta-analysis indicating the clear protective benefits of saffron.

The pathophysiology of chronic respiratory conditions such as asthma and COPD reveals that oxidative stress is caused by an imbalance in oxidant/antioxidant factors (Ghobadi et al., 2022b; Abedi et al., 2023). In asthma, oxidative stress is triggered by the excessive production of ROS by immune cells like eosinophils and neutrophils infiltrating the lungs (Albano et al., 2022). The ovalbumin-induced asthma model has been shown to result in elevated levels of MDA, a known oxidant factor, along with lowered levels of GSH, SOD, and catalase, which are all important antioxidant factors (Michaeloudes et al., 2022). The protective effects of saffron/its active ingredients have been revealed through their ability to enhance the balance between oxidants and antioxidants, offering another explanation for their mechanism of action.

The review article by Aslani et al. (2023b) thoroughly illustrates the impact of saffron on miRNA modulation. Elevated amounts of miR-146a and miR-106a have been observed in the ovalbumin-induced asthma model, with crocin playing a protective role through suppression of their expression (Aslani et al., 2022b). More research is needed to uncover how saffron and its active compounds influence the miRNA pathway on a mechanistic level.

Recently, one of the pathways of interest in inflammatory diseases such as asthma is the ER stress pathway. An increase in ER stress markers including ATF4, ATF6, CHOP, GRP78, and XBP1 has been detected in those with ovalbumin-induced asthma. Crocin has shown to protect against the expression of ER stress markers (Aslani et al., 2023b). While further studies are required, it seems that saffron might provide protection by regulating ER stress markers.

## 5 Limitation

One of the limitations of the current study was the use of different doses of saffron and its compounds, which was not analyzed based on concentration. On the other hand, the technique for using saffron varied among the studies, utilizing both oral ingestion and injections. Although ovalbumin injection and aerosol challenge were consistent methods across all studies, the variation in asthma induction duration was a notable limitation. The studys scope is limited by the chance of bias due to not incorporating all published studies into the analysis, given that some articles did not provide complete data. Another limitation in the study was found in the heterogeneity and variability in the experimental designs used across different studies.

## 6 Conclusion

The current comprehensive systematic review study showed for the first time that saffron and its active ingredients (crocin and safranal) have a protective function in animal studies of ovalbumin-induced asthma. The effects have been primarily demonstrated through various pathways such as anti-inflammatory, antioxidant mechanisms, modulation of ER stress markers, reducing AHR, and modulating inflammatory mediators.

## Data Availability

The original contributions presented in the study are included in the article/Supplementary Material, further inquiries can be directed to the corresponding author.

## Abbreviations

AHR: airway hyperresponsiveness, ATF4: activating transcription factor 4, ATF6: activating transcription factor 6, BALF: bronchoalveolar lavage fluid, CHOP: C/EBP homologous protein, COPD: Chronic obstructive pulmonary disease, EC50: half maximal effective concentration, GATA3: GATA Binding Protein 3, GRP78: glucose regulatory protein 78, GSH: glutathione, IFN-γ: interferon‐gamma, IgE: immunoglobulin E, iNOS: inducible nitric oxide synthases, I.P.: intraperitoneally, LDH: lactate dehydrogenase, IL: interleukin, MDA: malondialdehyde, miR: microRNA, NO: nitric oxide, NS: non-significant, OVA: ovalbumin, PCOS: polycystic ovary syndrome, SOD: superoxide dismutase, T-bet: T-box transcription factor, WBC: white blood cell, XBP1: transcription factor X-box binding protein.
