# Supplementary material for: Saffron (Crocus sativus) and its constituents in ovalbumin-induced asthma model: a preclinical systematic review and meta-analysis
Source: Front Pharmacol. 2024 Sep 4;15:1436295. doi: 10.3389/fphar.2024.1436295 (PMC11408349; doi:10.3389/fphar.2024.1436295)
Supplement: Supplementary file 1 [file DataSheet1.docx]

**Supplementary Files:**

| **Supplementary Table 1.** Search strategy in different databases. | | |
| --- | --- | --- |
| Databases | Search strategies | Number |
| PubMed | (“saffron”[tw] OR “crocus sativus”[tw] OR “saffron crocus”[tw] OR (crocus AND saffron) OR “crocin”[tw] OR “crocetin”[tw] OR “safranal”[tw] OR “picrocrocin”[tw])  AND  ("ovalbumin OR “OVA”[tw] OR “ova-sensitization”[tw] OR “ovalbumin sensitization”[tw] OR “ova sensitized”[tw] OR “ova-sensitized”[tw] OR “sensitized”[tw] OR “OVA-induced asthma”[tw])  AND  ("airway" OR “lung”[tw] OR “pulmonary”[tw] OR “asthma”[tw] OR “asthmatic”[tw] OR “Bronchial Asthma”[tw] OR (Asthma AND Bronchial) OR “lung remodeling”[tw] OR “pulmonary remodeling”[tw] OR “airways”[tw] OR “trachea”[tw] OR “respiratory”[tw] OR “allergic airway”[tw] OR “allergic asthma”[tw] OR “tracheal”[tw]) | 17 |
| Scopus | (TITLE-ABS-KEY("saffron" OR "saffrons" OR "crocus sativus" OR "saffron crocus" OR (crocus AND saffron) OR "crocin" OR "crocetin" OR "safranal" OR "picrocrocin")  AND  TITLE-ABS-KEY("ovalbumin" OR "OVA" OR "ova-sensitization" OR "ovalbumin sensitization" OR "ova sensitized" OR "ova-sensitized" OR “sensitized” OR “OVA-induced asthma”)  AND  TITLE-ABS-KEY("airway" OR "lung" OR "pulmonary" OR "asthma" OR “asthmatic” OR "Bronchial Asthma" OR (Asthma AND Bronchial) OR "lung remodeling" OR "pulmonary remodeling" OR "airways" OR "trachea" OR “respiratory” OR “allergic airway” OR “allergic asthma” OR “tracheal”)) | 16 |
| Web of sciences | (TS=("saffron" OR "saffrons" OR "crocus sativus" OR "saffron crocus" OR (crocus AND saffron) OR "crocin" OR "crocetin" OR "safranal" OR "picrocrocin")  AND  TS=("ovalbumin" OR "OVA" OR "ova-sensitization" OR "ovalbumin sensitization" OR "ova sensitized" OR "ova-sensitized" OR “sensitized” OR “OVA-induced asthma”)  AND  TS=("airway" OR "lung" OR "pulmonary" OR "asthma" OR “asthmatic” OR "Bronchial Asthma" OR (Asthma AND Bronchial) OR "lung remodeling" OR "pulmonary remodeling" OR "airways" OR "trachea" OR “respiratory” OR “allergic airway” OR “allergic asthma” OR “tracheal”)) | 21 |

| **Supplementary Table 2.** Quality assessment of included studies. | | | | | | | | | | | | | | | | | |
| --- | --- | --- | --- | --- | --- | --- | --- | --- | --- | --- | --- | --- | --- | --- | --- | --- | --- |
| **S/N** | **Study and year of publication** | **Q**  **1** | **Q**  **2** | **Q**  **3** | **Q**  **4** | **Q**  **5** | **Q**  **6** | **Q**  **7** | **Q**  **8** | **Q**  **9** | **Q**  **10** | **Q**  **11** | **Q**  **12** | **Q**  **13** | **Q**  **14** | **Q**  **15** | **Total score/15** |
| **1** | **Aslani (2021)** | **Yes** | **Yes** | **-** | **-** | **-** | **Yes** | **Yes** | **Yes** | **Yes** | **Yes** | **Yes** | **-** | **Yes** | **Yes** | **Yes** | **11** |
| **2** | **Aslani (2022)** | **Yes** | **Yes** | **-** | **-** | **-** | **Yes** | **Yes** | **Yes** | **Yes** | **Yes** | **Yes** | **-** | **Yes** | **Yes** | **Yes** | **11** |
| **3** | **Boskabady (2012)** | **Yes** | **Yes** | **-** | **-** | **-** | **Yes** | **Yes** | **Yes** | **Yes** | **Yes** | **Yes** | **-** | **Yes** | **Yes** | **-** | **10** |
| **4** | **Boskabady (2014)** | **Yes** | **Yes** | **Yes** | **-** | **-** | **Yes** | **Yes** | **Yes** | **Yes** | **Yes** | **Yes** | **-** | **Yes** | **Yes** | **Yes** | **12** |
| **5** | **Bukhari (2015)** | **Yes** | **Yes** | **-** | **-** | **-** | **Yes** | **Yes** | **Yes** | **Yes** | **Yes** | **Yes** | **-** | **Yes** | **Yes** | **Yes** | **11** |
| **6** | **Bayrami (2012)** | **Yes** | **Yes** | **Yes** | **-** | **-** | **Yes** | **Yes** | **Yes** | **Yes** | **Yes** | **Yes** | **-** | **Yes** | **Yes** | **-** | **11** |
| **7** | **Byrami (2013)** | **Yes** | **Yes** | **Yes** | **-** | **-** | **Yes** | **Yes** | **Yes** | **Yes** | **Yes** | **Yes** | **-** | **Yes** | **Yes** | **-** | **11** |
| **8** | **Gholamnezhad (2013)** | **Yes** | **Yes** | **-** | **-** | **-** | **Yes** | **Yes** | **Yes** | **Yes** | **Yes** | **Yes** | **-** | **Yes** | **Yes** | **-** | **10** |
| **9** | **Lertnimitphun (2021)** | **Yes** | **Yes** | **Yes** | **-** | **-** | **Yes** | **Yes** | **Yes** | **Yes** | **Yes** | **Yes** | **-** | **Yes** | **Yes** | **-** | **11** |
| **10** | **Mahmoudabady (2013)** | **Yes** | **Yes** | **Yes** | **-** | **-** | **Yes** | **Yes** | **Yes** | **Yes** | **Yes** | **Yes** | **-** | **Yes** | **Yes** | **Yes** | **12** |
| **11** | **Vosoghi (2013)** | **Yes** | **Yes** | **Yes** | **-** | **-** | **Yes** | **Yes** | **Yes** | **Yes** | **Yes** | **Yes** | **-** | **Yes** | **Yes** | **Yes** | **12** |
| **12** | **Xiong (2015)** | **Yes** | **-** | **Yes** | **-** | **-** | **Yes** | **Yes** | **Yes** | **Yes** | **Yes** | **Yes** | **-** | **Yes** | **Yes** | **Yes** | **11** |
| **13** | **Yosri (2017)** | **Yes** | **-** | **Yes** | **-** | **-** | **Yes** | **Yes** | **Yes** | **Yes** | **Yes** | **Yes** | **-** | **Yes** | **Yes** | **Yes** | **11** |

**Modified Tool:** (Q1) publication in a peer-reviewed journal; (Q2) statement of temperature control; (Q3) random allocation to groups (Q4) allocation concealment (Q5) blinded assessment of outcome; (Q6) description of the test methods (Q7) Description/origin of the intervention/venom (Q8) reports on the dose/concentration of venom and intervention (Q9) appropriate animal/test model (Q10) appropriate control group/test as part of the method (Q11) report of exposure period (Q12) sample size calculation; (Q13) reports of statistical methods employed (Q14) compliance with animal welfare regulations; (Q15) statement of potential conflict of interests.

| **Supplementary Table 3.** Risk of Bias in included studies. | | | | | | | | | | | | | | |
| --- | --- | --- | --- | --- | --- | --- | --- | --- | --- | --- | --- | --- | --- | --- |
| **S/N** | **Study and year of publication** | Query 1 | Query 2 | Query 3 | Query 4 | Query 5 | Query 6 | Query 7 | Query 8 | Query 9 | Query 10 | Query 11 | Query 12 | RISK |
| **1** | **Aslani (2021)** | **Yes** | **Yes** | **Yes** | **-** | **Yes** | **-** | **-** | **-** | **Yes** | **Yes** | **Yes** | **Yes** | **low** |
| **2** | **Aslani (2022)** | **Yes** | **Yes** | **Yes** | **-** | **Yes** | **-** | **-** | **-** | **Yes** | **Yes** | **Yes** | **Yes** | **low** |
| **3** | **Boskabady (2012)** | **Yes** | **Yes** | **Yes** | **-** | **Yes** | **-** | **-** | **-** | **Yes** | **Yes** | **Yes** | **Yes** | **low** |
| **4** | **Boskabady (2014)** | **Yes** | **Yes** | **Yes** | **Yes** | **Yes** | **-** | **-** | **-** | **Yes** | **Yes** | **Yes** | **Yes** | **low** |
| **5** | **Bukhari (2015)** | **Yes** | **Yes** | **Yes** | **-** | **-** | **-** | **-** | **-** | **Yes** | **Yes** | **Yes** | **Yes** | **medium** |
| **6** | **Bayrami (2012)** | **Yes** | **Yes** | **Yes** | **Yes** | **-** | **-** | **-** | **-** | **Yes** | **Yes** | **Yes** | **Yes** | **low** |
| **7** | **Byrami (2013)** | **Yes** | **Yes** | **Yes** | **Yes** | **Yes** | **-** | **-** | **-** | **Yes** | **Yes** | **Yes** | **Yes** | **low** |
| **8** | **Gholamnezhad (2013)** | **Yes** | **Yes** | **Yes** | **-** | **Yes** | **-** | **-** | **-** | **Yes** | **Yes** | **Yes** | **Yes** | **low** |
| **9** | **Lertnimitphun (2021)** | **Yes** | **Yes** | **Yes** | **Yes** | **Yes** | **-** | **-** | **-** | **Yes** | **Yes** | **Yes** | **Yes** | **low** |
| **10** | **Mahmoudabady (2013)** | **Yes** | **Yes** | **Yes** | **Yes** | **Yes** | **-** | **-** | **-** | **Yes** | **Yes** | **Yes** | **Yes** | **low** |
| **11** | **Vosoghi (2013)** | **Yes** | **Yes** | **Yes** | **Yes** | **Yes** | **-** | **-** | **-** | **Yes** | **Yes** | **Yes** | **Yes** | **low** |
| **12** | **Xiong (2015)** | **Yes** | **Yes** | **Yes** | **Yes** | **Yes** | **-** | **-** | **-** | **Yes** | **Yes** | **Yes** | **Yes** | **low** |
| **13** | **Yosri (2017)** | **Yes** | **Yes** | **Yes** | **Yes** | **-** | **-** | **-** | **-** | **Yes** | **Yes** | **Yes** | **Yes** | **low** |

**Query 1:** Was the allocation sequence adequately generated and applied? **Query 2:** Were the groups/test sample similar at baseline or homogenous? **Query 3:** Was the allocation adequately concealed or homogenous test samples? **Query 4:** Were the animals randomly housed during the experiment? **Query 5:** Were the test specimen appropriately and uniformly stored during the experiment? **Query 6:** Were the investigators blinded from knowing which intervention each animal/test group received during the experiment? **Query 7:** Were animals selected at random for outcome assessment? **Query 8:** Was the outcome assessor-blinded? **Query 9:** Were all major outcomes reported? **Query 10:** Are reports of the study free of selective outcome reporting? **Query 11:** Was the study apparently free of other problems that could result in a high risk of bias? **Query 12:** Was the administered dose uniform or the concentration level of the intervention homogenous?

**
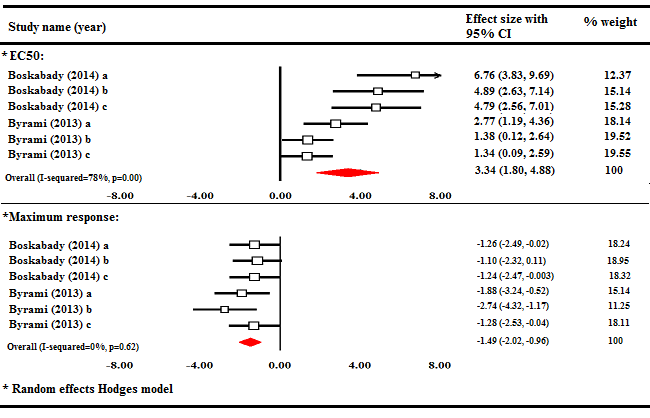
**

**Supplementary Fig. 1.** Forest plot detailing standardized mean differences (SMD) and 95% confidence intervals (CIs) in the studies reporting the effect of Crocus sativus, and Safranal on EC50 and maximum response values in intervention groups compared to OVA-induced asthma group. EC50: half maximal effective concentration.


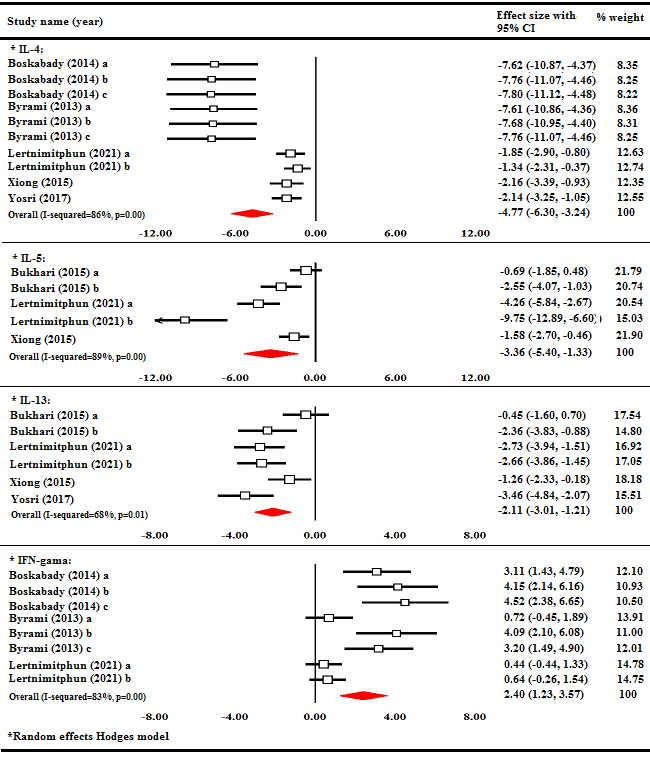


**Supplementary Fig. 2.** Forest plot detailing standardized mean differences (SMD) and 95% confidence intervals (CIs) in the studies reporting the effect of Crocus sativus, Crocin, and Safranal on inflammation parameters such as IL-4, IL-5, IL-13, and IFN-γ in intervention groups compared to OVA-induced asthma group. IL: interleukin, IFN-γ: interferon-gamma, OVA: ovalbumin.


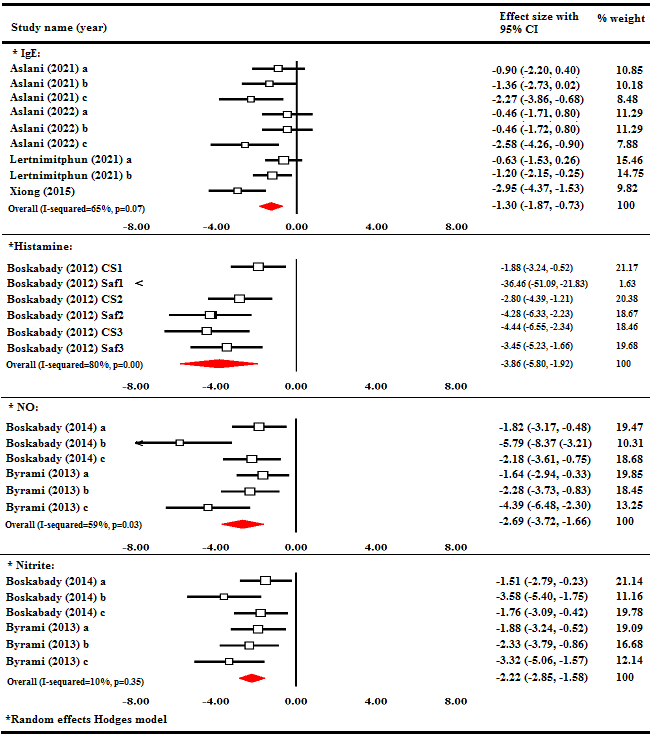


**Supplementary Fig. 3.** Forest plot detailing standardized mean differences (SMD) and 95% confidence intervals (CIs) in the studies reporting the effect of Crocus sativus, Crocin, and Safranal on inflammation mediatirs such as IgE, histamine, NO, and nitrite in intervention groups compared to OVA-induced asthma group. IgE: immunoglobulin E, NO: nitric oxide, OVA: ovalbumin.

**
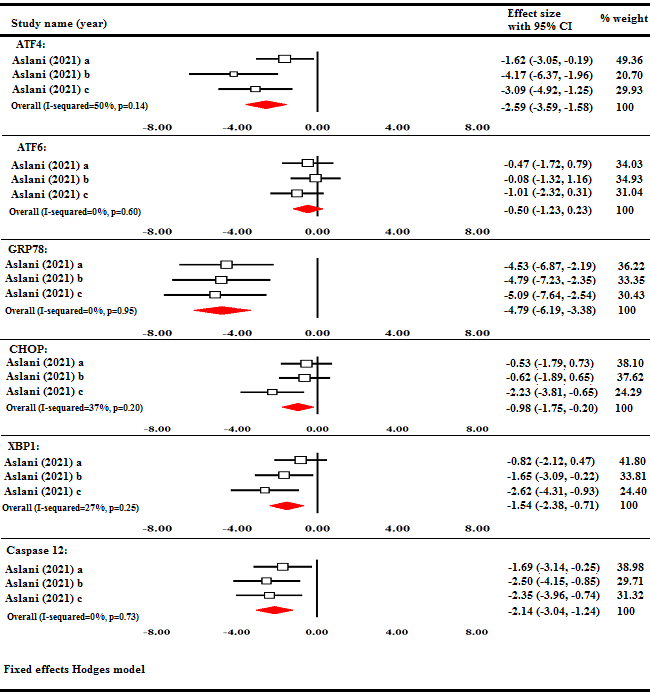
**

**Supplementary Fig. 4.** Forest plot detailing standardized mean differences (SMD) and 95% confidence intervals (CIs) in the studies reporting the effect of Crocin on ER stress such as ATF4, ATF6, XBP1, CHOP, GRP78, and caspase 12 in intervention groups compared to OVA-induced asthma group. ATF4: activating transcription factor 4, ATF6: activating transcription factor 6, CHOP: C/EBP homologous protein, GRP78: glucose regulatory protein 78, XBP1: transcription factor X-box binding protein, OVA: ovalbumin.

**
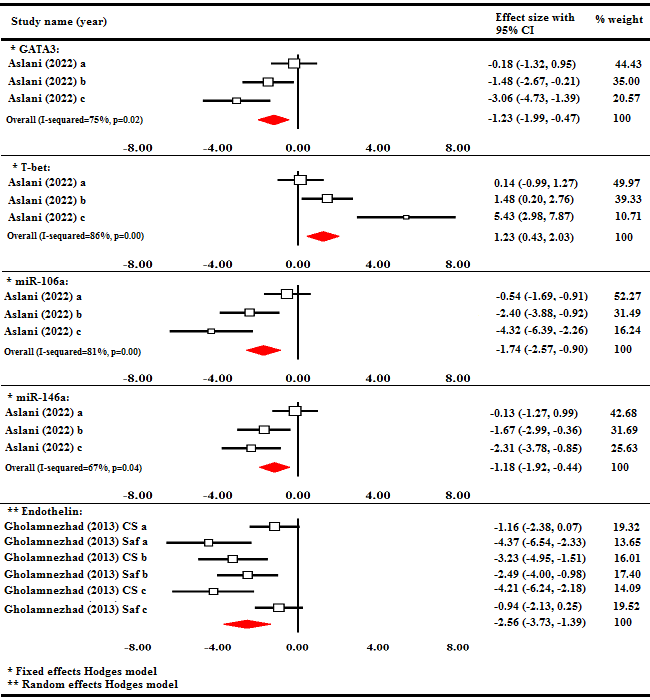
**

**Supplementary Fig. 5.** Forest plot detailing standardized mean differences (SMD) and 95% confidence intervals (CIs) in the studies reporting the effect of Crocin on GATA3, T-bet, miR-146a, miR-106a, and endothelin in intervention groups compared to OVA-induced asthma group. GATA3: GATA Binding Protein 3, T-bet: T-box transcription factor, miR: microRNA, OVA: ovalbumin.

**
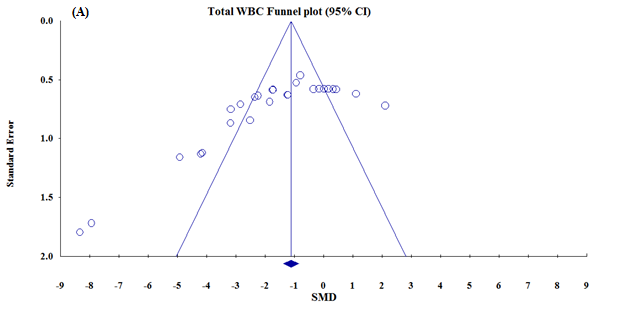
**

**
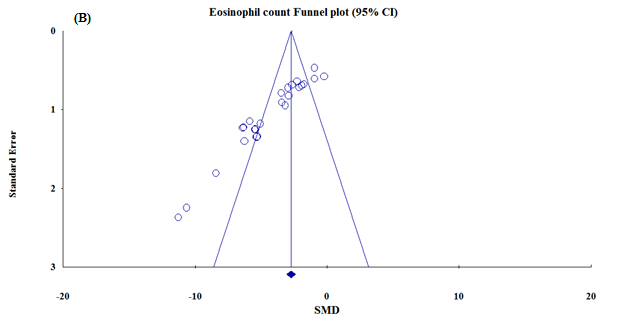
**

**
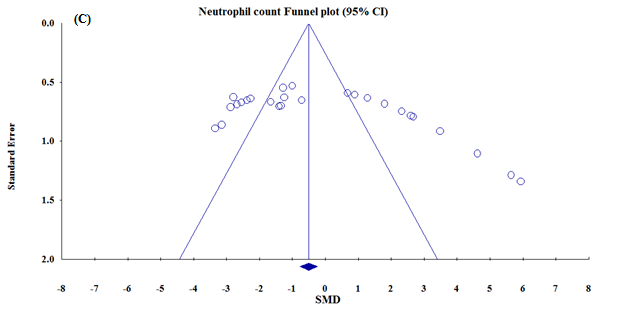
**

**
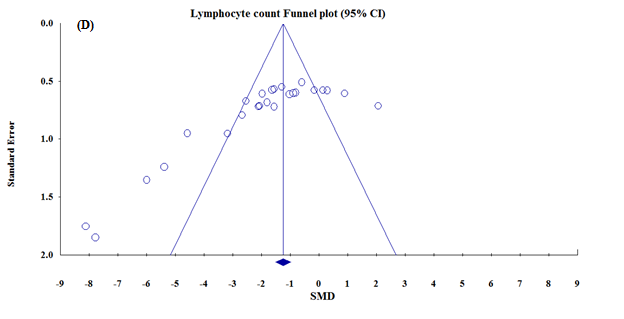
**

**
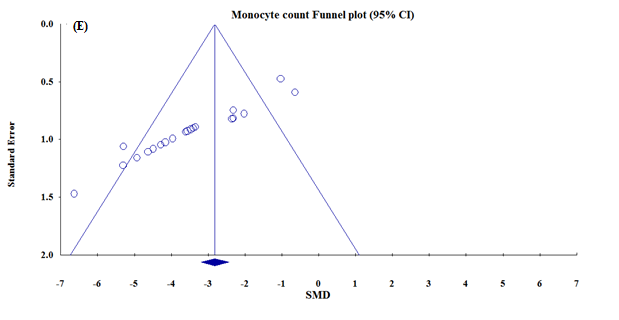
**

**
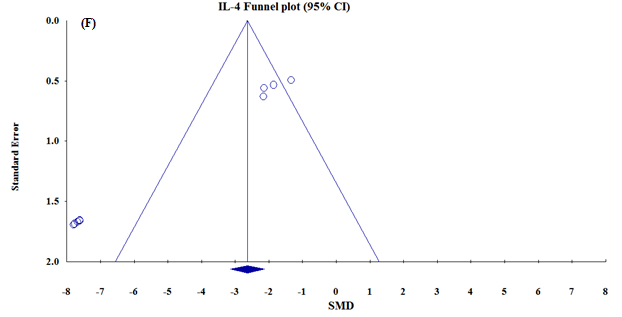
**

**
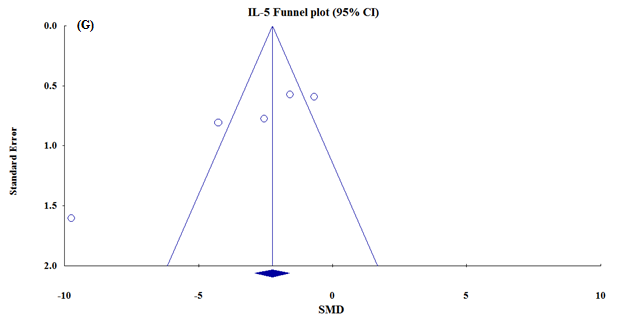
**

**
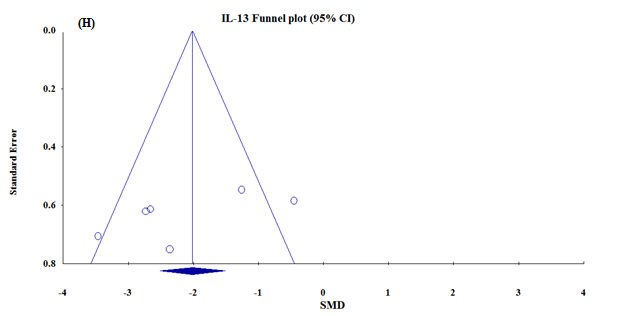
**

**
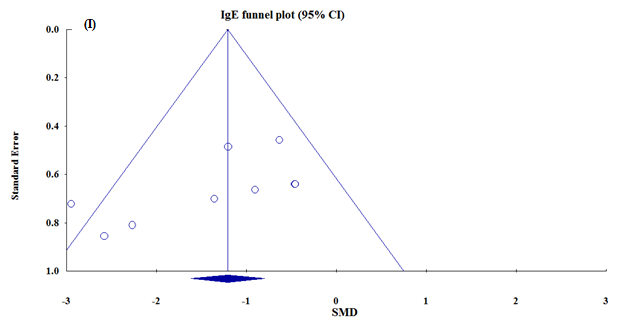
**

**
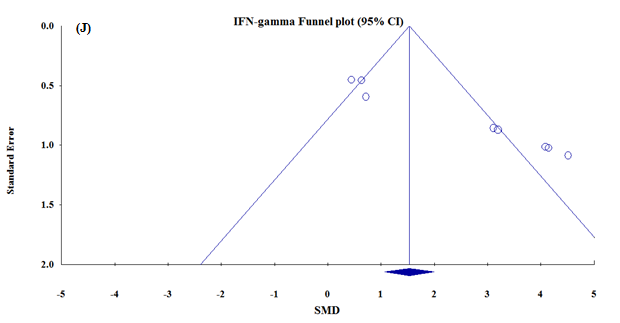
**

**Supplementary Fig. 6.** Funnel plot displaying the publication bias in the studies reporting the effect of saffron and its constituents on total WBC (A), eosinophil count (B), neutrophil count (C), lymphocyte count (D), monocyte count (E), IL-4 level (F), IL-5 level (G), IL-13 level (H), IgE level (I), and IFN-γ level (J) of treated and control animals.
